# Supplementary material for: Are carbon emissions trading and green financial instruments synergistic? -Comprehensive quantitative research based on content analysis
Source: PLoS One. 2024 Mar 7;19(3):e0298601. doi: 10.1371/journal.pone.0298601 (PMC10919601; doi:10.1371/journal.pone.0298601)
Supplement: S1 Data — (ZIP) [file pone.0298601.s001.zip › Data/地级市Z_F工作报告.zip!/╣π╬≈/╬α╓▌╩╨/└┤╫╘╣1⁄2╓┌║┼í╛┬φ┐╦╩2╛▌═°í┐_2014.docx]

各位代表：

　　现在，我代表市人民政府，向大会作政府工作报告，请予审议，并请各位政协委员和列席会议的同志提出意见。

2013年工作回顾

　　过去一年，在自治区党委、政府和市委的正确领导下，我们认真贯彻落实党的十八大和十八届二中、三中全会精神，以“三年一工程”为抓手，凝心聚力，攻坚克难，全力稳增长、调结构、强后劲、促改革、惠民生、保稳定，有效推动了经济社会发展实现稳中有进、稳中向好，较好完成了市十三届人大四次会议确定的各项目标任务。

　　——综合经济实力再上新台阶。全市完成地区生产总值991.7亿元，增长13.2%，增速排全区第2位；

全社会固定资产投资850.3亿元，增长30.6%，增速排全区第3位；

财政收入118.2亿元，增长17%，增速排全区第1位；

外贸进出口总额17.6亿美元，增长45.9%，增速排全区第1位。

　　——工业主导地位进一步显现。工业经济总量稳居全区第4位，完成规模以上工业总产值1745.5亿元、规模以上工业增加值581.5亿元，分别增长27.4%和20.2%，增速分别排全区第1位和第3位，工业对经济增长的贡献率达77%。荣获 “广西工业产业发展奖”一等奖。

　　——城市发展潜力显著提升。行政区划调整工作顺利完成，城市建成区面积拓展到90平方公里。苍海新区成为自治区重点推进的四大城市新区之一。我市被确定为自治区四大城镇群之一的桂东南城镇群核心城市。

　　——黄金水道建设成效初显。梧州港口货物吞吐量达到3015万吨，增长16%，占广西内河港口货物吞吐量的28%；集装箱货物吞吐量39.2万标箱，增长28%，占广西内河港口集装箱吞吐量的70%，广西内河港口集装箱吞吐量第一大港地位继续巩固。

　　——循环经济发展走在全国前列。初步建立以再生铜、再生铝、再生不锈钢、再生塑、再生棉为主的循环经济产业体系，循环经济产业总产值达到800亿元，我市成为广西首个国家级循环经济示范城市和新能源示范城市。

　　——城乡居民收入较快增长。城镇居民人均可支配收入22537元，增长9.6%；农民人均纯收入7475元，增长13.4%。

一年来，我们主要做了以下几项工作：

以项目建设为重点，深入实施“三年一工程”。坚持开展“项目攻坚年”活动，持续掀起项目建设新高潮。全市1004个重点项目完成投资828亿元，占全社会固定资产投资的98%。

坚持开展“服务企业年”活动，支持和引导企业转型发展。全市110家重点企业完成工业总产值946.8亿元，占规模以上工业总产值的54.1%，产值超亿元的企业达68家。

坚持开展“园区建设（招商）年”活动，推动产城融合发展。全市工业园区完成基础设施投资25亿元，累计落户企业672家。

坚持实施“安居乐业”工程，开工建设保障性住房10269套，建成5328套，分配入住3444套；完成农村危房改造8799户，一批低收入群体和困难群众实现了“安居梦”；城乡新增就业10.2万人，城镇登记失业率为3.9%；新发展微型企业2550户。

　　以振兴工业为重点，加快推进经济发展“三步棋”。召开全市工业发展大会，出台鼓励政策措施，推动工业转型发展。优先发展千亿元、百亿元产业，再生资源、再生不锈钢制品、电子信息和陶瓷产业迅速发展壮大，四大产业产值占全部工业总产值的比重超过64%。

加大技改力度，推动传统产业转型升级，实施技改项目280项，完成投资342亿元。大力推动科技创新，强化科技的支撑引领作用，建成了一批国家级高新技术企业和自治区级研发（研究）中心。

加快现代商贸物流业发展，全力推进市商贸物流园、毅德商贸物流城、中恒医药物流城、旺城广场、特色商业街等重点项目建设，100个在建商贸物流项目完成投资94.2亿元，增长29.2%。全市实现社会消费品零售总额292.3亿元，增长13.7%。

　　以发展特色农业为重点，加强和巩固农业基础地位。全市完成农林牧渔总产值187.9亿元，增长5%。多措并举力促粮食稳产增收，粮食总产量达83.9万吨，与全区同步实现八连增。

大力推进西江经济带梧州特色农业示范区建设，六堡茶、特色水果、中草药等特色优势种植业发展壮大，“梧州砂糖桔”获得国家地理标志产品保护认证；猪、鸡、兔、鱼、蜂等规模养殖业加速发展，畜禽标准化规模养殖场发展到151家；林下经济产值突破50亿元。

大力推进农业产业化经营，市级以上农业产业化重点龙头企业发展到91家。大力提高农业生产经营组织化水平，农民专业合作社发展到1180家。

探索新农村建设新模式，突出抓好一批示范点建设，全区新农村建设现场会在我市召开。加强防洪、病险水库除险加固、农村饮水安全、农田水利等工程建设，完成水利水电投资15.5亿元。

　　以生态宜居为重点，大力唱响城市建设“三部曲”。 创建国家园林城市各项考评指标全部达标，荣获国家园林城市称号。完成第三届广西园林园艺博览会（南宁）梧州园建设和开园工作，成功获得2015年第六届广西园林园艺博览会承办权。

基本完成行政区划调整工作，一县两区各项工作实现平稳过渡，城市发展空间进一步拓展。

大力推进城市新区开发建设，红岭新区基本成型，路网工程、中央商务区、玫瑰湖景观提升工程等项目稳步推进；苍海新区核心区加快建设，完成投资15.2亿元，苍海公园、西北片区岸线景观等项目建成。

加快重点城镇、特色城镇建设，岑溪“一河两岸”中心区和藤县河东新区建设稳步推进，蒙山特色旅游名县和苍梧六堡特色旅游名镇建设初见成效。

深入实施“绿满八桂”造林绿化工程，完成造林33.5万亩、村屯绿化67个，森林覆盖率提高到75.6%。

狠抓节能减排，规模以上工业万元增加值能耗下降9.3%，环境空气质量优良率、江河水质达标率均保持100%。

全面开展“美丽梧州·清洁乡村”活动，整合各类资金20.3亿元，新建了一大批乡镇、村庄垃圾中转站和垃圾池，农村生产生活环境有了较大改善。

　　以粤桂互联互通为重点，加快交通基础设施建设。南广高铁梧州段即将通车运营，梧州将迎来高铁时代。

岑溪至罗定高速公路全线通车，梧州至贵港、梧州至柳州、岑溪至水汶、市环城等高速公路项目加快推进。梧州机场迁建项目完成立项前期工作。

西江黄金水道建设加快，李家庄码头三期工程试运营，紫金村综合码头、中储粮码头主体工程完工，大利口码头开工建设，长洲水利枢纽三线四线船闸加快建设，区域性综合联运港口地位日益显现。

着力完善西江沿江路网，龙圩至潭东公路“二改一”工程、梧州西江机场进场道路等项目进展顺利，藤州至长洲三龙二级公路、207国道东绕城过境一级公路等项目前期工作加快推进。

大力实施农村公路建设攻坚大会战。全市交通基础设施建设完成投资53.8亿元，占年度计划投资的105%。

　　以向东开放为重点，不断提高开放合作水平。积极实施“东向”战略，全面推进与粤港澳的交流合作，主动承接东部产业转移，加快向先进生产力靠拢，承接产业转移的规模不断扩大、层次明显提升，各产业园区特色鲜明，发展态势良好。

深化梧肇战略合作，召开第二次梧肇战略合作市长联席会议，组织实施26项重点项目和重大事项，完成投资23.6亿元；推动梧云战略合作，与云浮市签署《梧州—云浮战略合作协议》；

全力加快粤桂合作特别试验区建设，先行区形成“三园一城”的发展格局，起步区的道路、桥梁等基础设施建设快速推进，试验区进驻企业38家，实现规模以上工业总产值94亿元。

成功举办第十届梧州国际宝石节和第六届西江经济发展论坛，提升了城市知名度和影响力。

　　以改善民生为重点，推动社会各项事业协调发展。加大民生投入，市财政在民生方面的支出达138.2亿元，占公共预算支出的78.5%。

12件民生实事如期完成，投资额达44.4亿元。

完善社会保障体系，实现城乡居民社会养老保险制度全覆盖，实际参保人数达116.3万人，基础养老金标准提高到每人每月75元；连续第九年调整提高企业退休人员养老待遇，调整后月人均养老金提高到1679.5元；发放城乡低保金3.6亿元，城乡低保月人均补助金额增长10%；

出台了被征地农民社会保障实施办法，进一步保障了被征地农民的利益。

保持物价基本稳定，居民消费价格指数低于控制目标。

加大179个贫困村整村推进扶贫开发力度，帮助近6万贫困人口实现脱贫。

突出抓好教育民生工程，筹措3.56亿元实施了农村中小学危旧校舍改造等项目，筹措8100万元资助11.6万多困难学生；农村义务教育寄宿制学校贫困学生营养改善计划惠及12万多学生，1.43万名进城务工人员随迁子女全部实现就近入学。

深入推进医改工作，全民医保体系初步建立，全市三项基本医疗保险参保率均达98%以上；就医报销实现即时结算，703个村卫生室实行“1元钱”看病。

丰富群众文体生活，成功举办或承办全国龙狮大联动广场体育活动、全区粤剧邀请赛、全市首届社区文艺汇演等大型文体活动，积极推进广西第十三届运动会的各项筹备工作。

加快农村广播电影电视公共服务体系建设，实施农村电影公益放映工程，放映场次达10391场。

稳步推进人口和计划生育工作，全面完成自治区下达的各项责任目标任务。设立村组、社区法律援助工作站138个，为1539名群众提供了法律援助。

加强和创新社会管理，积极推行社区网格化管理模式，实现社区服务管理全覆盖。扎实开展安全生产大检查和环境风险排查整治行动，安全生产形势总体稳定。

深入推进法治梧州、平安梧州建设，大力开展以藤县为重点的社会治安综合整治，有力维护了社会和谐稳定。

　　以转变作风为重点，切实加强政府自身建设。认真执行市人大及其常委会的决议、决定，主动接受市人大的法律监督、工作监督和市政协的民主监督，人大代表建议和政协委员提案办结率100%。建立健全政府绩效考评机制，推动政府重大事项集体决策、科学决策、民主决策。以“三力”建设为抓手，加强公务员队伍建设，不断提高行政效能。加强政风行风建设，深入开展“两大评议”活动，坚决整治“庸懒散奢”等问题。加强审计监督，对全市20个部门、单位领导开展任期经济责任审计。全面推进政务公开、网络问政工作。深化行政审批制度改革，共取消、下放和调整行政审批项目269项。扩权强县、食品药品监管体制、财政预决算公开和“营改增”试点等改革工作扎实推进。加强政务服务中心建设，推动政务服务向基层延伸，全市80%的乡镇（街道）和30%的行政村（社区）建立了政务服务中心。进一步转变文风会风，以市政府名义印发的文件和召开的会议均较上年减少。严格执行中央八项规定，坚决反对“四风”，严控“三公”经费支出，从简办节办会。

　　一年来，中直、区直驻梧各单位为推动梧州发展作出了不懈努力和积极贡献。编制、法制、民族、宗教、外事、侨务、对台、统计、物价、国资、供销、地震、人防、农机、移民、住房公积金、房改办等工作取得了新进展。工会、共青团、妇女儿童、残联、老龄、科协、社会科学、慈善、档案、地方志等工作取得新进步。国防教育和后备力量建设深入开展，双拥共建工作取得新成绩。

　　各位代表！过去一年，我们面对的形势复杂严峻，取得的成绩来之不易，这是自治区党委、政府和市委正确领导的结果，是市人大及其常委会、市政协有效监督和大力支持的结果，是全市人民攻坚克难、共同奋斗的结果。在此，我代表市人民政府，向全市各条战线上的干部群众，向人大代表、政协委员，向各民主党派、工商联、无党派人士、人民团体，向离退休老领导、老同志，向驻梧部队、武警官兵、公安干警，向所有关心支持梧州发展的社会各界人士，表示崇高的敬意和衷心的感谢！

　　在肯定工作和成绩的同时，我们也清醒地认识到政府的工作以及经济社会发展中还存在一些深层次的矛盾和问题，主要是：经济长期平稳较快发展的基础还不牢固，内生动力仍然不足；改革开放的广度和深度还不够，制约发展的体制机制障碍有待进一步突破；财政和城乡居民持续增收困难加大，民生工作离群众的要求还有差距；资源环境约束加大，节能减排任务艰巨，安全生产工作基础仍较薄弱；行政区划调整后续工作依然繁重；政府职能有待进一步转变，行政效能有待进一步加强，“四风”问题还需下力气整治等。这些问题，我们将采取更加有力的措施认真加以解决，决不辜负人民群众的期望！

2014年工作总体要求和主要目标

　　今年是深入贯彻落实党的十八届三中全会精神、全面深化改革的关键之年，也是全面完成“十二五”规划的攻坚之年。总体上看，今年的经济发展环境要好于去年，但面临的形势仍然较为复杂，特别是项目融资、银行信贷紧张的局面仍将持续，环境倒逼机制下转方式、调结构的压力也会更大，生产性商贸物流、旅游服务业发展相对滞后等自身积累的结构性矛盾还没有很好解决，全面深化改革的大背景下发展面临的竞争将更加激烈，前进的道路上不可避免的还会遇到各种困难和挑战。在这种复杂形势下，我们既要看到发展面临的不利因素，更要看到加快发展的难得机遇和有利条件。中央坚持稳增长、调结构、促改革，相继出台了一系列重大改革举措。国家建设21世纪“海上丝绸之路”，支持广西打造新的战略支点。自治区深入实施“双核”驱动战略，珠江—西江经济带建设即将上升为国家战略，梧州的区位优势进一步凸显，被自治区定位为西江黄金水道上向东开放的龙头城市和桂东南城镇群的核心城市。经过多年积累，我市奠定了良好的发展基础，构建了结构合理的现代工业体系，形成了“五园八区”的产业发展格局，打造了“五位一体”的立体交通网络，创建了国家“城市矿产”示范基地、国家循环经济示范市、国家园林城市、国家低丘缓坡土地开发利用试点城市、桂东承接产业转移示范区等一批“国字号”发展平台。在新的起点上，我们必须抢抓机遇，乘势而上，不松劲、不懈怠，坚持发展第一要务不动摇，努力保持高于全国全区平均水平的增长速度；必须坚持开放合作，主动对接先进生产力，大力承接产业转移，不断积蓄持续发展新动力；必须坚持改善民生，让人民群众过上更加幸福美好的生活；必须坚持保护生态环境，既要金山银山，也要绿水青山，实现经济、社会、生态全面协调发展。

　　今年政府工作的总体要求是：全面贯彻落实党的十八大和十八届二中、三中全会及自治区党委十届四次全会、市委十二届四次全会精神，坚持稳中求进的工作总基调，把改革创新贯穿于经济社会发展各个领域各个环节，加快转方式、调结构，切实提高经济发展的质量和效益，坚持“东向”战略，以“三年一工程”为抓手，继续走好经济发展“三步棋”，唱响城市建设“三部曲”，强工业、固基础、兴三产、促转型，加快推进新型城镇化，加强生态建设和环境保护，促进经济健康较快发展、社会和谐稳定，加快建成西江黄金水道上向东开放的龙头城市。

今年经济社会发展的主要预期目标是：地区生产总值增长11%，力争12%；全社会固定资产投资增长20%，力争25%；财政收入增长12%，力争13%；规模以上工业增加值增长15%，力争17%；社会消费品零售总额增长14%，居民消费价格总水平涨幅控制在4%左右，城镇居民人均可支配收入增长11%，农民人均纯收入增长12%，城镇登记失业率控制在4.5%以内，完成自治区下达的节能减排任务。

2014年工作安排

　　为确保实现今年的发展目标，努力促进经济社会持续健康较快发展，应重点做好以下工作：

　　一、强化项目支撑带动

　　坚持以“三年一工程”为抓手，把项目建设作为集聚生产要素、拉动投资增长、推动经济发展的关键，进一步优化投资结构，全力以赴推进项目建设。

　　打好项目攻坚战。创新开展“项目攻坚年”活动，加快建设一批大项目、好项目，以项目大开发、大建设推动经济社会大发展、大进步。今年全市安排重点项目1105项，总投资3436亿元，年度完成投资836.8亿元，其中续建项目489项，计划完工425项，年度完成投资494.4亿元；计划新开工项目526项，年度完成投资341.4亿元；安排重点前期项目90项，总投资632.1亿元。

　　大力开展招商引资。深入开展“招商引资年”活动，保持招商引资良好势头。充分利用国际国内两种资源、两个市场，突出面向粤港澳地区，紧盯世界名企、央企和实力民企，深入推进“央企入梧”、“民企入梧”和“金企入梧”。

围绕再生资源、电子信息、新材料、新能源、商贸物流、生态养生等重点产业，以及粤桂合作特别试验区、梧州工业新城等重点园区，积极开展合作招商、商（协）会招商以及产业联盟招商等。以产城融合为重点，突出抓好县域招商。发挥专业招商队的作用，主动对接、紧密跟踪，全力加快签约项目落户步伐。

力争全市招商引资实际到位资金673亿元，增长16%。

　　强化项目要素保障。进一步加强政银企对接，争取金融机构加大对重点项目、重点企业的信贷投放力度，积极拓宽中小企业融资渠道；不断规范政府融资平台公司管理，提高直接融资比重，保障项目建设资金及时到位，强化募集资金监管，切实防范债务风险。积极争取项目建设用地指标，用好用足用活低丘缓坡等土地综合开发政策，提高土地节约集约利用率，加大闲置土地处置力度，切实保障项目建设用地。完善项目联合审批制度，把握国家财政、货币和产业政策走向，加快项目前期工作。深入总结推广一线工作法、协同工作制、项目前期代办制等一系列办法措施和工作经验，深化拓展“五个一”跟踪服务制度，通过抓项目、促管理、建制度，实现项目建设加速跑。

　　二、推进工业跨越发展

　　坚持雁行阵势梯次布局产业发展，围绕“3年突破3千亿，5年跨越5千亿，8年超过1万亿”的工业发展总体目标，以存量强根基，以增量促调整，大力培育千亿元、百亿元产业集群，提高工业发展的质量和效益。

　　加快产业转型升级。优先发展再生资源、再生不锈钢制品和电子信息等3个千亿元产业，加快推进20万吨光亮铜杆、金海不锈钢二期、永达不锈钢四期等重点项目；

改造提升食品、医药、机械制造、林产林化、日用化工等传统优势产业，推进神冠三期、中恒新药扩产等300项技改项目，力争完成技改投资400亿元。

加快发展新一代信息技术、新型材料、节能环保等战略性新兴产业，突出抓好广西微软创新中心和IT学院落户梧州，重点推进鑫华通科技园、盛意达电子文化创意城等一批项目建设，支持惠普（中国）、深商集团等知名企业在梧州设立分公司或生产基地。

深入实施质量兴梧和品牌战略，集中力量培育一批具有国际国内竞争力的品牌企业。

加快推进科技创新体系建设，承办好第四届广西发明创造成果展览交易会。

　　促进园区扩容提质。以加强园区交通、供水、供电等基础设施，以及教育、文化、医疗等配套设施建设为重点，加快“五园八区”提质扩容。坚持产业发展和城镇建设“一张图”规划，加快梧州工业新城基础设施建设，高位推进新城规划调整和项目前期工作。以引进龙头企业为主攻方向，以完善产业配套为助推器，加快电子信息产业园区建设，形成园区经济新的增长极。加快推进岑溪新材料产业园建设，着力构建梧州新材料产业体系。推进宝石集中加工园区规划建设。力争园区完成基础设施投资28亿元，完成工业项目投资155亿元，实现工业总产值1900亿元；

建成产值达500亿元以上园区1个，300亿元以上园区2个，百亿元以上园区6个。

　　扶持企业做大做强。坚持扶优扶强，继续选择110家关联性强、成长性好、发展潜力大的工业企业进行重点扶持，在用地保障、财税支持、能源资源倾斜等方面出台政策措施，努力为企业解决实际问题，力争产值百亿元以上的工业企业达4家、50亿元以上6家、10亿元以上30家、亿元以上280家。

大力发展中小企业，发挥大企业的示范引领作用，引导企业注重质量品牌建设，提高核心竞争力，在市场竞争中不断发展壮大。

降低市场准入门槛，拓宽投融资渠道，加大培育企业上市力度，对处于种子期、初创期的科技型、创新型中小企业进行专项资金扶持。

贯彻落实全区非公经济发展大会的决策部署，积极营造良好的创业环境，进一步激发非公经济的创新活力和发展动力，努力形成“大企业顶天立地，中小企业铺天盖地”的格局。

　　三、扎实做好“三农”工作

　　按照中央农村工作会议部署，全面落实强农惠农富农政策，深化农村改革，夯实农业基础，促进现代农业加快发展、农村更加繁荣、农民持续增收。

　　强化农业基础地位。稳定粮食播种面积和产量，确保粮食播种面积239万亩以上，粮食总产达到82万吨，每个县（市）建立一个平均亩产达到500公斤以上的千亩超级稻示范样板片，促进粮食增产，保障粮食安全。加强农业基础设施建设，重点推进江河堤防、水库除险加固、中小河流治理、农村饮水安全、农田水利等工程建设，进一步改善农村生产生活条件。加快发展农业机械化，大力推广农业新机具、新技术，不断提升农机装备水平。加大农业科技投入，抓好农产品质量安全监管和动植物重大疫病防控，发挥科技支撑作用，促进农业可持续发展。

　　推进农业现代化。加快构建以农民家庭承包经营为基础、合作与联合为纽带、社会化服务为支撑的现代农业经营体系，培育发展家庭农场、专业大户、产业化龙头企业和农民专业合作组织。

加快推进西江经济带梧州特色农业示范区建设，大力实施柑桔上山优果工程，推进六堡茶集中加工区、1.2万亩“菜篮子”和品牌蔬菜基地以及高脂松、油茶标准化基地的建设，加快发展生猪、三黄鸡、养兔、养蜂等特色养殖业，努力提高农业发展多元化、规模化、集约化水平。

加快农产品流通体系建设，推进农超、农校、农企对接。加强示范、突出特色，积极推进新农村建设。

　　拓宽农民增收渠道。深入实施农民收入“倍增计划”，建立促进农民增收的长效机制；着力提高农业生产效益，促进家庭经营收入稳定增长；加快引导农村劳动力转移就业，促进工资性收入持续增加；加大对农业的补贴力度，促进转移性收入大幅增长；积极稳妥推进农村改革，创造条件增加农民的财产性收入；深化扶贫攻坚，加大整村推进扶贫开发工作力度，实施精准扶贫，再推动一批贫困人口实现脱贫致富。让农民通过自己的辛勤劳动，也能进入小康。

　　四、提速发展现代服务业

　　今年我市将迈进高铁时代，高速度、高密度、公交化的高铁运行方式，让服务业最先最直接受益。要以此为契机，大力发展以现代服务业为主的第三产业，努力提高第三产业占地区生产总值的比重。

　　加快发展商贸物流业。进一步完善物流园区、货运站场、配送中心等物流节点与各港口、产业园区的互联互通，积极引进和培育现代物流企业，着力构建多层次的物流载体。统筹推进重点商贸流通项目119项，完成投资105.6亿元。加快推进市商贸物流园、毅德商贸物流城、东盟农副产品交易中心等一批重点商贸物流园区，以及苍梧旺甫农副产品物流园、岑溪泽仁商贸城、藤县河东商贸中心、蒙山万村千乡物流中心等一批大型商贸物流项目的建设。

继续推进特色商业街建设，促进商贸物流与旅游、文化等产业融合发展。推动物流信息平台和标准化建设，完成梧州口岸货运船检码头改造工程，营造良好通关环境。

积极推广物流标准化体系应用，不断提高物流运作管理水平。

加快发展旅游、养生等产业。围绕建设旅游目的地和游客集散地，加快构建“一核两带四区”旅游发展新格局。大力实施旅游建设大会战，统筹安排重点旅游项目103项，完成投资43.21亿元。

整合资源，加大投入，支持蒙山县率先创建特色旅游名县，推动苍梧六堡旅游特色名镇建设实现新突破。加强对历史文化的挖掘，谋划两广总督府、岭南首府、舜帝文化公园、古钱鉴遗址、广信楼等历史文化项目，着力打造一批旅游支撑品牌。创建国家3A级以上景区2家、三星级以上饭店4家、乡村旅游区4家、星级农家乐1家，力争全市旅游总收入达133亿元以上。

统筹推进城乡养老服务业发展，引导社会资本投入养老机构建设，建立电子智慧居家养老服务信息系统，创建全国老年友好型城市。依托良好的生态资源和岑溪、蒙山“中国长寿之乡”的品牌资源，培育发展富有特色的生态养生产业，抓好岭南生态养生城招商、选址、基础设施建设等工作。

　　培育发展新兴服务业。加快建设大型综合商业中心、特色街区、县级商业中心，大力发展电子商务、连锁经营和物流配送等现代流通业态。结合城市新区开发和旧城改造，优化商业网点结构和布局，进一步拓展便民直销店进街道、进社区，力争全年实现社会消费品零售总额333亿元以上。积极发展科技服务、信息服务、现代金融、文化创意、会展和服务外包等新兴产业。引入物联网和云计算技术，建设梧州“核心网络通道”、“互联网数据中心”、“智慧城市”、“信息基地高速接入”等四大工程。

　　五、着力推进新型城镇化

　　围绕建设桂东南城镇群核心城市，优化城镇布局，增强中心城市辐射带动能力，加快构建产城融合、城乡协同的新型城镇体系。

　　加快中心城市建设。按照“一江两岸三组团”的城市发展思路，统筹推进城市新区开发建设和旧城改造工作，提高城市综合承载能力和辐射带动功能。加大红岭新区及其拓展区开发建设力度，突出抓好路网工程、西江三桥、中央商务区、体育场等项目建设；大力推进苍海新区核心区建设，重点抓好半岛核心区、环城水系、环湖道路、园博园、南广高铁站前广场等项目建设；加快南岸片区开发，抓好西江四桥及道路、供水、排水、供电等基础设施建设，启动沿江景观大道规划研究。继续加大旧城改造力度，加快平民冲、冰泉冲和石鼓冲的开发建设；把棚户区改造纳入保障性安居工程，全力推进实施。启动长洲岛整体开发规划研究工作。

统筹抓好县城和小城镇建设。把发展中小城市、小城镇作为优化城镇结构的主攻方向，实施大县城战略，有重点地发展小城镇，加快构建中心城市、县城和小城镇合理分工、功能互补、协同发展的城镇体系。

扩大县城建成区面积，壮大经济实力，完善城市功能，积极吸纳人口，着力将岑溪、藤县培育为中等城市，加快新苍梧县城规划建设工作。

加快培育经济强镇、特色小镇，推进历史文化名镇、名村建设，突出抓好六堡、太平、归义等镇的规划建设。完善农村水、电、通信网络等配套设施，深入推进城乡风貌改造工程，推动市政设施向农村延伸、公共服务向农村覆盖、城市文明向农村辐射。坚持自愿、分类、有序原则，加快推进农业转移人口市民化。

　　加强城市规划管理。遵循“规划应适当超前，建设可分步实施”的理念，加快修订完善城市总体规划，提高规划水平，强化规划的刚性约束。

实施中心城区交通畅通工程，推进主次干道建设和重要节点改造，提升城区路网通达能力和通畅水平。加快公共停车设施建设，创新停车设施管理体制，切实解决城市停车难、停车乱的问题。深入开展占道经营、泥头车不规范装运等专项整治行动，加大“打非治违”工作力度，坚决遏制乱搭乱建和违规抢建等行为。

创新城市管理机制，进一步理顺市区两级市政设施管理体制，提升城市管理工作水平和市民满意度。

　　六、加快建设现代综合交通运输体系

　　坚持交通优先发展战略，以粤桂互联互通为重点，加快构建方便、快捷、外通内畅的现代综合交通运输体系。

　　加快完善综合交通运输枢纽。围绕建设粤桂交通大通道，大力推动南广铁路全线通车，加快岑罗铁路建设，促进柳梧铁路开工；推动环城高速公路全线大规模施工。围绕西江经济带建设，基本建成梧贵、岑水高速公路，加快梧柳高速公路建设，推动荔浦至玉林高速公路尽快开工；全力加快机场迁建步伐，力争项目开工建设；积极拓展航线，力争开通梧州至北京、深圳等航线。围绕沿江路网和重要节点联通，基本建成龙圩至潭东一级公路，加快建设207国道东绕城过境一级公路和西江机场进场道路，力争开工建设苍梧环镇公路六堡至狮寨段等项目，加快207国道石桥绕城公路、龙圩至赤水码头沿江一级公路等项目前期工作；

加大农村公路建设力度，实施农村公路安保工程，提升农村公路安全畅通水平。加快实施西气东输二线“县县通”工程，完善城镇管道燃气基础设施，提高天然气入户率。

　　加快建设区域性综合联运港口。建成长洲水利枢纽三线四线船闸、紫金村综合码头、中储粮码头、大利口码头一期等水运项目，实施桂江旺村枢纽坝下航道整治工程，全面推进赤水铁路专用线建设，

加快赤水圩作业区二期工程前期工作并适时启动建设。争取一类口岸扩大到赤水码头。

　　着力优化交通运输组织。完善中心城区至龙圩的公交线路，实现新老城区公交无缝衔接。

加快建设梧州南站综合客运枢纽、玫瑰湖汽车客运站和城北客运站。

深入开展城区交通秩序联合整治行动，加强出租汽车、公共汽车行业监管，优化城市公交线路和站点设置。

　　七、深入推进开放合作

　　坚定不移地推进“东向”战略，强化改革意识、开放意识和市场意识，以更大的力度向东开放，向东看齐，主动接受先进生产力的辐射带动。

　　全力建设粤桂合作特别试验区。按照珠江—西江经济带发展规划，加快编制试验区相关专项规划。

推动两省区成立联合领导小组，推进试验区管理体制机制和优惠政策先行先试，争取国家赋予突破性政策，破解试验区统一管理、规划、建设和利益共享等关键问题，积极探索和推动跨行政区划合作与开发管理的新机制、新模式。加快推进起步区基础设施建设，优化先行区产业布局，加快建设塘源一路、污水处理厂、产业规划馆等，促进落户项目尽早开工，推动试验区建设取得更大突破。

　　积极拓展多区域合作。充分发挥区位优势，进一步扩大对粤港澳的开放合作，推进与粤港澳建立合作交流机制。

深化与肇庆、佛山、云浮等地的战略合作，进一步完善合作沟通机制，在园区建设和项目引进、用地、融资、审批等方面实施优惠政策，提供全程优质服务，推进承接产业转移迈出更大步伐。以中国—东盟博览会为平台，

进一步扩大与东盟各国的合作交流。办好梧州国际宝石节、第十届中国茶业经济年会，拓展与区内外各市的合作交流。加大对外宣传力度，努力扩大梧州的知名度和影响力。

　　大力发展外向型经济。调整优化出口产品结构，努力巩固传统市场，大力开拓新兴市场，千方百计保持外贸出口稳定增长，重点扩大电子、松香、纺织、机电等产品的出口比重。加快口岸开放、查验机制等方面的创新，启动综合保税区申报工作，推动口岸通关一体化，提高货物进出口周转效率，降低进出口企业的物流成本，促进贸易投资自由化、便利化。力争全市外贸进出口总额达21亿美元。

　　八、全面加强生态建设

　　坚持走经济发展与环境保护双赢的道路，大力推动绿色发展，保住“山清水秀生态美”的金字招牌。

　　加快发展循环经济。牢固树立循环经济理念，大力推进循环型产业、企业、园区和社会体系建设，扎实推进国家循环经济示范市建设。以国家“城市矿产”示范基地建设为切入点，以再生园区、不锈钢制品园区为载体，加快构建产业链条完备的循环再生产业体系。启动生活垃圾处理、餐厨废弃物资源利用和无害化处理、医疗废弃物处置、污水处理厂剩余污泥处置等项目的集中规划建设工作。大力发展生态循环型农业，积极推广林业生物质能源及“养殖—沼气—种植”三位一体的能源生态发展模式，提高林业产业剩余物利用率。

　　打造生态宜居环境。深入实施“绿满八桂”造林绿化工程，力争完成植树造林面积20万亩，巩固森林覆盖率；大力推进环城山体低效公益林改造和沿江沿路及景区林相改造，使森林发展与生态旅游、林下经济发展相得益彰。

巩固创建国家园林城市成果，推进城市绿道、花园式街道建设，加大城市花卉栽养力度，提高园林绿化美化水平。全力以赴抓好第六届广西园博会筹备工作。

深入开展“清洁家园、清洁水源、清洁田园”专项行动，推动清洁乡村活动常态化，进一步改善乡村人居环境，提升乡村生态建设水平。

　　强化生态环境保护。实行最严格水资源管理制度，执行产业发展规划和环境保护规划，决不引进高污染、高排放、对生态环境压力大的项目，坚守生态环境保护红线和底线。深入开展环境安全隐患大清查大整治，加强农村环境连片整治，加大环境保护考核指标权重，实行环境信用评价。深入研究国家生态扶持政策，积极配合自治区推动两广合作开展小流域生态补偿试点，加大水生动物资源保护力度，确保沿江生态安全。

　　九、大力增进民生福祉

　　坚持把保障和改善民生作为一切工作的出发点和落脚点，继续实施一批惠民工程，加快推进公共服务均等化，让发展成果更多更公平地惠及全市人民。

　　继续实施为民办实事工程。为民办实事是履行党的宗旨、体现执政为民的重要举措，是各级政府义不容辞的责任。今年继续筹措资金42亿元以上，全力实施社保、健康、教育、强基、安居、土地整治、农补、生态、文体、交通、能源、消费等12项为民办实事工程。

　　加强就业和社会保障工作。加快推进“乐业工程”，重点做好高校毕业生、就业困难群体、农村转移劳动力等群体的就业工作，确保实现“三个100%”的目标，实现城乡新增就业9.3万人（次）以上，城镇登记失业率控制在4.5%以内，劳动力技能培训6万人以上，确保全市就业形势基本稳定。

加快完善城乡社会保障体系，扩大社会保险覆盖面，建立“五险合一”社保信息管理系统。

加快推动保障性安居工程，加大农村危房改造力度，确保完成11163套保障性住房建设任务。认真落实城乡最低生活保障标准等工作。

　　推动教育振兴发展。继续深化教育教学改革，统筹各级各类教育协调发展。以自治区实施“千亿元建千所学校”工程为契机，多渠道筹措资金新建、改扩建一批学校。进一步加大教师队伍建设力度。推进城乡义务教育资源均衡配置，重点实施农村中小学、幼儿园教师培训提升工程。加强普通高中基础能力建设，促进普通高中多样化发展。加快现代职业教育体系建设，深化产教融合、校企合作，提高职教的吸引力、服务力和毕业生留梧就业率。深入实施高等教育内涵提升工程，支持梧州学院加快发展，稳步推进更名为梧州大学的各项筹备工作。

　　统筹推进其他社会事业。全面实施卫生项目建设攻坚，启动市医疗养老护理中心项目，实施工人医院改扩建工程，完成市人民医院新院一期搬迁，加快乡镇卫生院周转房建设。巩固新型农村合作医疗制度，推进全市80%以上的村卫生室实现“一卡通”刷卡结算。完善生育政策，加强计生管理。加快完善公共文化服务体系，着力推进公共文化惠民、文艺精品创作、文化遗产保护、文化产业培育、“扫黄打非”等工作。加快实施广播电视“村村通”工程，力争广播电视综合覆盖率达到98%以上。

扎实推进广西第十三届运动会各项筹备工作，加快比赛场馆建设，加强运动员训练备战，完善公共体育服务体系，全面提升体育事业发展水平。

　　十、努力建设人民满意政府

　　必须突出转变作风，进一步加强政府自身建设，做好改革发展稳定各项工作。

　　加快职能转变。切实履行经济调节、市场监管、公共服务、社会管理、环境保护等职能，努力建设服务型政府。强化科学、民主、依法决策，健全决策机制、程序和决策问责、纠错制度。推进权力运行公开化、规范化，完善政务公开和各领域办事公开制度，促进阳光行政。健全行政执法责任制、过错责任追究制，严格规范公正文明执法。自觉接受人大法律监督和工作监督，接受政协民主监督，重视公众监督、舆论监督，加强审计监督。

　　改进社会治理方式。坚持依法治市，推进“法治梧州、平安梧州”建设。加强社区管理服务创新，激发社会组织活力，创新有效预防和化解社会矛盾体制，健全公共安全体系，提高社会治理水平。认真开展矛盾排查调处工作，完善人民调解与行政调解、司法调解衔接联动工作机制。不断完善防汛抗洪等应急预案，强化事故灾难、公共事件等预报预警，全面提升应急处置能力。加强社会管理综合治理，加大对各类违法犯罪的打击力度，不断提高人民群众的安全感和满意度。加强食品药品安全监管，确保群众吃得安全、吃得放心。严格落实安全生产责任制和责任追究制，以“零容忍”的态度、铁的手腕和严厉的措施，深入排查治理安全生产隐患，坚决遏制各类重特大事故。

　　推进重点领域改革。深化行政审批制度改革，做好行政审批事项“接、放、管”，对上级下放的行政审批事项，接好管好；对上级取消的行政审批事项，坚决取消；对市级仍需保留的行政审批事项，简化商事登记制度，依法依规管好。深化投融资体制改革，全面放宽投资领域，吸引更多的社会资金参与投资、建设、运营。深化农村改革，开展农村土地承包经营权确权登记，积极稳妥有序地推进土地集中流转，按照国家政策赋予农民对承包地占有、使用、收益、流转及承包经营权抵押、担保权能，允许农民以承包经营权入股发展农业产业化经营。推进医药卫生体制、食品药品监管体制改革，加大县级公立医院改革力度，巩固基本药物制度，完善基层运行新机制。

　　全面加强作风建设。严格执行中央八项规定、党政机关厉行节约反对浪费条例、国内公务接待管理规定等各项禁令，坚决做到令行禁止，确保政令畅通。深入开展党的群众路线教育实践活动，以深化“三力”建设为载体，进一步改进作风，坚决反对“四风”、整治“六病”，树立为民务实清廉新形象。深入推进惩治和预防腐败体系建设，加强政风行风建设，围绕群众关心的热点问题，深入开展专项治理，严肃查处各类违纪违法案件，纠正损害群众利益的不正之风，不断提高政府公信力和执行力。树立科学的政绩观，把民生改善、社会进步、科技创新、生态效益等指标和实绩作为考核干部政绩的重要依据。大力弘扬进取精神、实干精神、担当精神、创新精神，始终保持昂然向上、奋发有为的精神状态，以踏石留印、抓铁有痕的劲头狠抓各项工作的落实。

　　各位代表，在全力抓好以上重点工作的同时，我们还要广泛开展双拥共建活动，加强国防动员、国防教育和国防后备力量建设；依法保障公民特别是妇女、未成年人和残疾人的合法权益，充分发挥工会、共青团、妇联等群团组织的作用；继续做好外事、对台、民族、宗教、统计、人防、气象、地震、水文、档案、地方志等工作。

　　各位代表，做好今年经济社会发展各项工作，任务繁重，使命光荣，责任重大。让我们紧密团结在以习近平同志为总书记的党中央周围，在自治区党委、政府和市委的坚强领导下，锐意进取，团结拼搏，扎实工作，奋力完成今年各项目标任务，为加快实现富民强梧新跨越，加快建成西江黄金水道上向东开放的龙头城市而努力奋斗！
